# A Machine Learning-Based Case–Control Study on Suicide Risk Identification: Integrating Acoustic and Linguistic Features Under Stress Conditions

**DOI:** 10.1155/da/1671972

**Published:** 2025-08-08

**Authors:** Qunxing Lin, Jianqiang Zhang, Weijie Wang, Chunxin Tan, Xiaohua Wu, Jiubo Zhao

**Affiliations:** ^1^Digital Mental Health and Risk Identification and Control Lab, Department of Psychology, School of Public Health, Southern Medical University, Guangzhou, China; ^2^Department of Psychiatry, Zhujiang Hospital, Southern Medical University, Guangzhou, China

**Keywords:** acoustic features, machine learning, stress-inducing conditions, suicide risk, word frequency features

## Abstract

Suicide is a significant global public health issue, with current risk assessment methods primarily relying on psychiatrists' clinical judgment and scale-based evaluations, which can be challenging to implement. Recently, interest has increased in using vocal and linguistic features to identify suicide risk. This study investigates speech-based methods for assessing suicide risk in two phases involving 90 patients with major depressive disorder (MDD) or bipolar disorder (BD). In Phase 1, three types of question-answer materials with different emotional valences (positive, neutral, and negative) were employed. The model combining acoustic and word frequency features from negative emotional valence materials achieved the highest accuracy at 77.82%. Phase 2 introduced stress factors, highlighting that speech data collected under stress better reflects participants' psychological states, providing more insights into suicide risk. These findings emphasize the potential of speech analysis in suicide prevention, while also calling for further research to validate and expand these results.

**Summary**

• What is already known on this topic: Suicide risk assessment has traditionally relied on clinical judgment and scale-based evaluations, which can be difficult to implement and may lack accuracy. There is increasing interest in utilizing vocal and linguistic features to assess suicide risk, particularly among individuals with mental health disorders such as major depressive disorder (MDD) and bipolar disorder (BD).
• What this study adds: This study explores the use of speech-based methods to assess suicide risk in patients with MDD or BD, through a two-phase evaluation. It demonstrates that combining acoustic and word frequency features, especially from speech produced under negative emotional conditions, offers the highest accuracy in identifying suicide risk. Additionally, incorporating stress factors in Phase 2 revealed that speech data collected under stress provides a more accurate reflection of psychological states, thereby, enhancing the identification of suicide risk.
• How this study might affect research, practice or policy: These findings highlight the potential of speech analysis as a tool for suicide risk assessment and underscore the need for further research to validate these results. The approach could lead to the development of more accurate, objective tools for identifying suicide risk, improving early detection and intervention in clinical settings, and influencing policies related to mental health and suicide prevention.

## 1. Introduction

Suicide is one of the most pressing public health challenges worldwide. According to the World Health Organization (WHO), over 700,000 individuals died by suicide in 2019, accounting for more than one in every 100 deaths. Suicide is currently the fourth leading cause of death among individuals aged 15–29 [1]. In China, adolescents and young adults—particularly those aged approximately 12–35 years—have been identified as a high-risk population for suicide [2]. Recent statistics indicate that approximately 200,000 individuals in China die by suicide each year, with a suicide rate of about 8.3 per 100,000 people [3]. An age-period-cohort analysis of data from 2004 to 2019 revealed a rising trend in suicide risk among those aged 10–34 years [4]. Suicide is often considered a common pathogenic behavior resulting from mental illnesses, especially depressive disorder and bipolar disorder (BD) [5]. An epidemiological study indicates that individuals with BD are 15 times more likely to attempt suicide compared to the general population [6]. Furthermore, in patients with major depressive disorder (MDD), the suicide attempt rate is about 16.3%, and the suicide mortality rate is approximately 4.1%, highlighting a significantly higher suicide risk for individuals with MDD during their lifetime [7]. During the follow-up period, BD patients who experienced depressive episodes for a higher proportion of time showed a significant association with increased suicide risk [8]. A study explored the risk factors for suicidal ideation and attempts among patients with depressive and BDs, finding that MDD is an independent predictor of suicidal ideation, particularly in the absence of psychotic symptoms [9]. These studies collectively suggest that patients experiencing depressive episodes are a high-risk group for suicide. Understanding the suicide risk during these episodes is crucial for suicide prevention efforts.

In addition to mental illnesses, suicide is influenced by various other factors, with stress being one of the most closely related and impactful [10]. It has been noted that mental health issues are primary risk factors for suicide, while psychosocial factors, such as acute stress events, are secondary risk factors [11]. Research has demonstrated that an acute stress response is a significant risk factor for suicide, especially when it co-occurs with other mental health issues such as depression or substance abuse, which can further increase the risk [12]. A study found that acute stress is a significant predictor of suicidal behavior in patients with acute transient psychotic disorder (ATPD). Identifying this risk factor helps clinicians better recognize and manage suicide risk in ATPD patients, particularly during psychiatric emergencies [13]. Another study investigated the transition from suicidal ideation to attempts among adolescents and found that an increased number of recent stressful life events significantly heightened suicidal risk. Interpersonal conflicts and losses related to intimate relationships, such as romantic breakups and the death of close friends or family members, were particularly indicative of increased risk for suicide attempts [14]. Furthermore, a case-cohort study revealed that the presence of stress disorders significantly increased the rate of suicide attempts. Individuals diagnosed with any stress disorder had a suicide attempt rate nearly 13 times higher than that of the control cohort [15]. It is crucial to pay significant attention to assessing suicide risk in individuals who have experienced stressful events.

Currently, suicide risk assessment primarily involves clinical evaluations by psychiatrists, who utilize various scales such as the Beck Scale for Suicide Ideation, the Columbia-Suicide Severity Rating Scale (C-SSRS), and the Ask Suicide-Screening Questions (ASQ) [16–18]. These assessments, combined with the patient's medical history and the clinician's own experience, are used to evaluate and judge the patient's condition. Despite the transition from using a single scale to incorporating multiple scales and considering their combined results, identifying suicide risk remains challenging [19]. Some patients subjectively conceal their suicidal thoughts or symptoms, particularly those who intentionally hide their true intentions to end their lives, posing significant obstacles to suicide prevention and intervention efforts [20, 21]. Unlike many medical fields that rely on objective and reproducible biomarkers for diagnosis, the assessment of suicide risk still heavily depends on the patient's voluntary disclosure of information and the observations and judgments of healthcare professionals [11]. Objective markers for suicide risk diagnosis are still under exploration.

In recent years, many researchers have continually attempted to identify potential diagnostic tools for suicide risk. Language and speech, as critical data sources for diagnosing mental disorders, have attracted significant research interest due to their low collection cost, noninvasive nature, and the potential to simultaneously extract acoustic and word frequency features during speech collection [22]. A study collected recordings from 104 patients with mood disorders through clinical interviews and extracted acoustic features, achieving a 69% accuracy rate in identifying suicide risk [23]. Voice samples collected via the internet were used to extract acoustic features, demonstrating the feasibility of predicting depression severity and screening for suicidal ideation using these features [24]. Research analyzing word usage on internet forums related to depression, anxiety, and suicidal thoughts found a trend of “absolutism” in language, with individuals or potential patients more frequently using polarized words such as “always” and “never” [25]. And a review study proposed that suicidal ideation can be predicted through the use of more intensifiers, while suicidal behavior can be predicted through changes in pronoun and verb usage, increased use of prepositions, and fewer modifiers and numbers [26].

Although researches have made some progress, it still has limitations, such as overreliance on single acoustic or word frequency features and the collection of speech data primarily from clinical interviews. To enhance the model's generalizability and accuracy, a series of innovative measures was implemented. First, the variety of research materials was enriched by incorporating question-and-answer materials with three different emotional valences: positive, neutral, and negative. This strategy aims to broaden the coverage of speech data to more comprehensively capture acoustic and word frequency features related to suicide risk. In Phase 1, speech data corresponding to three emotional valences were gathered and employed to build the preliminary model. The comparison of these data facilitated the identification of the most effective emotional valence materials and the model best suited for detecting suicide risk. This screening process not only refined the model's ability to identify risks but also yielded insights into the correlation between speech features across various emotional states and the potential for suicide risk. Subsequently, the study incorporated stress factors, drawing on the optimal valence materials and models identified. In some cases, individuals may find themselves in highly stressful conditions, providing only speech data. For illustration, the National Center for Mental Health and mental health prevention in China provides a 24-h telephone counseling service, a key component of suicide prevention efforts. Under these circumstances, counselors depend on callers' speech data to evaluate suicide risk, a process vital for precise assessment of crisis scenarios and formulation of effective response strategies. The in-depth analysis of these data sought to elucidate the patterns of speech characteristic changes under stress and to refine the model for enhanced accuracy in identifying suicide risk across varying stress levels.

## 2. Methods

### 2.1. Objective

The primary objective of this study is to develop and evaluate a suicide risk identification model that integrates acoustic and word frequency features, assessing their effectiveness under stress conditions in patients with MDD or BD. The study is structured in two phases. The first phase aims to construct and compare suicide risk identification models based on acoustic features, word frequency features, and their combination, using question-and-answer materials with varying emotional valences (positive, neutral, and negative). The second phase introduces stress-inducing factors to explore whether stress-elicited speech data more accurately reflects participants' psychological states, thereby, highlighting the potential of speech analysis in suicide prevention and informing future research directions.

### 2.2. Study Size

**Figure 1:**
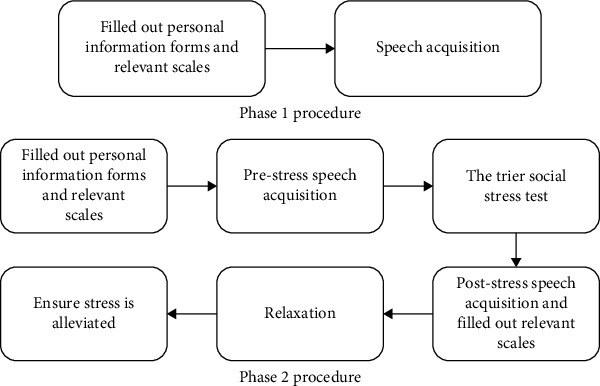
Experimental flowchart.

The study was conducted in two phases, each with a distinct sample size based on the specific objectives and design of the phase. In the first phase, 60 participants were recruited to develop and evaluate the initial suicide risk identification models using acoustic and word frequency features. In the second phase, 38 participants were recruited to assess the impact of stress factors on speech data and its ability to better reflect psychological states. The sample sizes for each phase were determined through power analysis using G*⁣*^*∗*^ Power 3.1.9.7. For the first phase, the significance level (*α*) was set at 0.05, with a desired power (1 − *β*) of 0.80, assuming an effect size (Cohen's *d*) of 0.5, and accounting for a 10% nonresponse rate. Similarly, the second phase followed a tailored power analysis, reflecting the specific research objectives and practical considerations. These sample sizes were designed to ensure adequate statistical power within each phase, although the relatively modest size limits the generalizability of findings. Future research could benefit from larger and more diverse samples to enhance external validity. The experimental process is shown in Figure 1.

### 2.3. Patient and Public Involvement

Patients and the public were not involved in the design, conduct, or reporting of this study. The research focused on participants with MDD or BD, and their involvement was limited to data collection and analysis related to suicide risk identification through speech features. No formal patient or public advisory groups were consulted during the development of the research or the manuscript.

### 2.4. Participants

Ninety-eight patients (60 in Phase 1 and 38 in Phase 2) with MDD or BD were recruited from Zhujiang Hospital in Guangdong Province. Participants ranged in age from 12 to 35 years and demonstrated fluency in Mandarin, and no participants dropped out. In Phase 1, 60 participants were enrolled, including 40 females; in Phase 2, 38 participants were enrolled, including 27 females. Diagnoses were made by a senior psychiatrist according to the criteria outlined in the Diagnostic and Statistical Manual of Mental Disorders, Fifth Edition (DSM-5) criteria. To further validate the diagnoses, all participants were assessed using the Mini International Neuropsychiatric Interview (MINI) and the Young Mania Rating Scale (YMRS). The exclusion criteria were as follows: (1) history of organic brain diseases; (2) presence of a current severe psychological crisis; (3) electroconvulsive therapy within the 3 months preceding the study; (4) ongoing manic symptoms; (5) severe psychotic symptoms; (6) language impairment or disorder.

Ethical approval was granted by the Biomedical Ethics Committee at Southern Medical University (Approval No: NFYKDX003, Approval Date: May 2022). All participants or their guardians agreed to participate in this study and signed a written informed consent form.

## 3. Materials

Drawing from pertinent literature and established scales, a set of questions was extracted, structured, and refined to comprise eight items per emotional valence (negative, neutral, and positive), culminating in a total of 24 potential questions. This collection of questions was then formatted into a questionnaire and distributed via the Wenjuanxing platform using a convenience sampling approach. Participants assessed each question on a 1-to-9 scale, rating both pleasantness and arousal, where 1 indicating extremely unpleasant or unaroused, and 9 signified extremely pleasant or aroused. The mean and standard deviation of the ratings for pleasantness and arousal across these questions were calculated.

The questions were organized by arousal level, ranging from low-to-high. For Phase 1, three questions were selected each for each valence category: positive, negative, and neutral. During Phase 2, the selection process focused on four negative valence questions. Comprehensive details regarding the questions are provided in the Supporting Information (Materials S1, S2).

## 4. Measure

### 4.1. Trier Social Stress Test (TSST)

The TSST is a widely recognized laboratory protocol designed to induce psychosocial stress in experimental settings [27]. It typically consists of a 10-minute anticipation phase followed by a 10-minute test phase, during which participants are required to deliver a speech and perform mental arithmetic tasks in front of an evaluative audience [28]. The TSST reliably provokes robust physiological stress responses, including elevated cortisol levels and increased heart rate. It has been extensively employed to examine various dimensions of stress reactivity, such as gender differences, developmental trajectories, and genetic moderating effects [29]. Due to its flexibility, the TSST provides a valuable framework for investigating the interplay between social-evaluative threat, interpersonal processes, and neurophysiological stress mechanisms [30]. Modified versions of the TSST have also been developed for group administration, use with children, and integration with neuroimaging techniques, further enhancing its utility in psychobiological research [29].

### 4.2. Self-Compiled Basic Information Questionnaire

The questionnaire includes personal information, encompassing ethnicity, gender, age, educational background, only child status, marital status, disease diagnosis, and medication history. It also collects supplementary information on family residence, family financial status, family history of mental illness, family history of suicide, and history of suicide among acquaintances.

### 4.3. MINI-Chinese Version

The MINI is a structured interview tool developed to assess 16 Axis I psychiatric disorders according to the DSM-V and ICD-10 diagnostic criteria [31]. The Chinese adaptation demonstrates high reliability and validity within the domestic context, comprising 130 items, and is used for the identification and diagnosis of mental disorders [32]. The suicidality module of the MINI is widely regarded as a validated tool for evaluating suicide risk. The module employs a four-tiered scoring system for suicide risk, ranging from no risk (0 points) to low-risk (1–5 points), medium risk (6–9 points), and high-risk (10 points or above). In this study, participants who scored in the no risk or low-risk categories (0–5 points) were categorized into the low suicide risk group, whereas those with medium or high-risk (6 points or higher) were classified as the high suicide risk group.

### 4.4. YMRS

The YMRS is a widely utilized examiner-rated scale for the evaluation of manic symptoms, with high reliability and validity [33]. Comprising 11 items, the YMRS indicates the presence of mild or more severe manic symptoms when the score exceeds five. The scale is principally employed to screen out participants exhibiting manic symptoms.

### 4.5. Patient Health Questionnaire Depression Scale (PHQ-9)

The PHQ-9 is widely recognized as a frequently utilized self-report scale for the assessment of depression across both domestic and international settings [34]. The reliability and validity of PHQ-9 have been extensively conformed in the Chinese context. This study employs the PHQ-9 to evaluate the current depressive symptoms of the participants. The Cronbach's *α* coefficient for the PHQ-9 was 0.91 in Phase 1 and 0.92 in Phase 2, indicating high internal consistency.

### 4.6. Suicidal Behaviors Questionnaire-Revised (SBQ-R)

The SBQ-R comprises four items and serves as a convenient tool for identifying suicide risk. Scores exceeding seven on the SBQ-R typically suggest the likelihood of suicide risk [35]. The Cronbach's *α* coefficient for the SBQ-R was 0.86 in Phase 1, and 0.83 in Phase 2.

### 4.7. Beck Scale for Suicide Ideation-Chinese Version (BSI-CV)

The Beck Scale for Suicide Ideation (BSSI) is extensively applied in evaluating the intensity of suicidal thoughts [36]. It consists of 19 items, with a total score of 38 points, where higher scores denote a greater severity of suicidal ideation. For this study, the Cronbach's *α* coefficient was 0.92 in Phase 1, and 0.97 in Phase 2.

### 4.8. Short State Anxiety Inventory (SSAI)

The State-Trait Anxiety Inventory (STAI) is a concise tool designed for the rapid assessment of anxiety levels [37]. In this study, the SSAI was used during Phase 2 to evaluate at baseline, following the TSST, and after the relaxation task. The Cronbach's *α* coefficients for this scale were as follows: 0.80 at baseline, 0.78 after the TSST, and 0.75 following the relaxation intervention.

### 4.9. The Positive and Negative Affect Schedule (PANAS)

The PANAS evaluates current emotional states through 20 items that gauge both positive and negative affect [38]. In the present study, only the negative affect subscale of PANAS was employed in Phase 2 to gauge participants' stress responses at baseline, post-TSST and following the relaxation task. The Cronbach's *α* coefficients for the negative affect subscale exceeded 0.89 at all time points.

## 5. Procedure

The study protocol encompassed two distinct phases conducted separately. During Phase 1, participants completed personal information forms and relevant scales, which included the PHQ-9, SBQ-R, and BSI-CV. Following a 5-min rest, participants progressed to the speech acquisition stage of Phase 1, during which they verbally responded to nine questions posed by examiners, with microphones clipped to their collars.

In Phase 2, the TSST was used to provoke acute stress in a controlled laboratory setting. The TSST is recognized as a prevalent protocol for inducing stress, capable of generating high-intensity stress within a relatively brief timeframe. To maintain uniformity in the experimental process, a comprehensive “Examiner Manual” and “TSST Interviewer Guidelines” were developed before the experiment. All examiners and interviewers underwent centralized training and additional reinforcement based on pre-experiment results.

Participants completed the scales enumerated in Phase 1 before the experiment, and additional scales including the SSAI and PANAS at baseline, after the TSST, and after the relaxation task. Speech acquisition in Phase 2 occurred twice separately before and twice after the TSST, with each session consisting of four questions. The Latin square design was implemented to equitably distribute the order of the questions across all sessions. Following a 10-min mindfulness breathing exercise with an audio guide, participants were assessed to confirm the alleviation of their stress levels. If needed, a therapist will provide additional relaxation training to participants until they reach a state of calm (Figure 1).

Speech data acquisition was conducted in soundproof, electromagnetically shielded, and independent laboratories, where ambient noise levels were maintained below 40 dB to create optimal conditions for the capture of high-quality speech recordings. The raw audio files, initially captured in AVI format, were converted to WAV format for subsequent acoustic feature extraction and analysis. Throughout the data processing, recordings with excessive noise or segments missing were excluded to ensure data quality.

## 6. Feature Extraction

The current study employed Librosa, a Python-based toolkit for audio and music signal processing, to manage voice signals and extract critical acoustic features [39]. Librosa offers a range of functions that facilitate the extraction of features including fundamental frequency (F0), shimmer, zero-crossing rate, sound intensity, loudness, energy, formants, Mel-frequency cepstral coefficients (MFCCs), linear prediction coefficients (LPCs), linear prediction cepstral coefficients (LPCCs), and line spectral pairs (LSPs). Additionally, long-term statistical features, such as maximum, minimum, median, mean, variance, skewness, and kurtosis were calculated for each segment of the voice recordings.

The study adopted the Chinese version of the Linguistic Inquiry and Word Count (LIWC) dictionary [40–42] which encompassed categories such as overall descriptive words, language process words, psychological process words, and personal concern words. The “Wenxin Chinese Psychological Analysis System,” developed by the Computer Network Psychological Laboratory at the Institute of Psychology, Chinese Academy of Sciences, was used to extract linguistic features and to compute the frequency percentage of words across various categories. Lexical features were collected from the recordings according to the specifications of this dictionary.

### 6.1. Statistical Analyses and Classification

Statistical analyses were conducted using SPSS 26.0. The Shapiro–Wilk test was conducted to access the normality assumption of the data distribution. Given the non-normal distribution of acoustic features and word frequency features, a nonparametric Mann–Whitney *U* test was employed to compare the high and low suicide risk groups. Demographic data were analyzed using the Chi-square test, while scale data were examined with the independent sample *t* test. The stress effect was evaluated through analysis of variance (ANOVA).

Based on literature review, the random forest algorithm was selected for model construction, supported by its demonstrated superior performance in various studies [43–45]. The random forest model was developed and evaluated using a five-fold cross-validation approach. Model performance was evaluated based on metrics including accuracy, the area under the ROC (AUC), and the F1 score. All machine learning model training and evaluation processes were conducted using Scikit-learn, a Python-based toolkit. To ensure model comparability, the random forest parameters were predefined: n_estimators was set to 200, the criterion was the GINI index, and random_state was set to 0. Following the nonparametric test, significant features were incorporated into the model construction process.

## 7. Results

### 7.1. Results of Phase 1

The MINI suicidality risk assessment module classified 38 participants into the low suicide risk group and 22 into the high suicide risk group. Demographic data analysis revealed no statistically significant differences between the two groups across key variables (all *p* > 0.05). It is worth noting that previous research has identified gender as a key factor influencing suicide risk, with females typically exhibiting higher levels of suicidal ideation and attempts. In the sample, the high-risk group also included more female than male participants; however, this difference did not reach statistical significance (*χ*^2^ = 0.144, *p*=0.705), possibly due to the limited sample size. A detailed overview of the demographic characteristics is provided in Supporting Information (Table S1). Independent samples *t*-tests yielded the following outcomes: Participants in the high suicide risk group exhibited significantly higher scores on the PHQ-9 (*t*[58] = 3.616, *p* < 0.001), the SBQ-R (*t*[58] = 5.564, *p* < 0.001), and the BSI-CV (*t*[58] = 4.344, *p* < 0.001) compared to those in the low suicide risk group. The YMRS scores suggested that neither group exhibited manic symptoms (*t*[58] = 0.183, *p*=0.092), aligning with the inclusion and exclusion criteria. Detailed descriptive statistics for each scale are provided in Supporting Information Table S2.

Analysis of the three suicide risk identification models developed from data collected in Phase 1 revealed that the model integrating both acoustic and word frequency features outperformed those that relied solely on either acoustic or word frequency features, achieving an accuracy rate of 77.82% (see Table 1). Consequently, the construction of subsequent models would incorporate both acoustic and word frequency features, and Phase 2 would be executed to investigate the influence of stress states on the identification of suicide risk.

### 7.2. Results of Phase 2

According to the MINI suicidality interview module, 12 participants in Phase 2 were classified into the low suicide risk group, and 26 participants were assigned to the high suicide risk group. Among those in the high-risk group, 18 were female and eight were male, again showing a higher number of females with elevated suicide risk. However, this gender difference was not statistically significant (*χ*^2^ = 0.133, *p*=0.715). No other demographic variables showed significant differences between the two groups (all *p* > 0.05). A detailed overview of the demographic characteristics is provided in Supporting Information (Table S3). Independent samples *t*-tests revealed that participants in the high-risk group exhibited significantly higher scores on the PHQ-9 (*t*[36] = 3.547, *p* < 0.001), the SBQ-R (*t*[36] = −11.52, *p* < 0.001), and the BSI-CV (t[36] = 3.527, *p* < 0.001) compared to those in the low-risk group (all *p* < 0.01). Additionally, the YMRS scores indicated that neither group displayed manic symptoms (*t*[36] = 0.502, *p*=0.619), which aligned with the inclusion and exclusion criteria. Detailed descriptive statistics for each scale are provided in Supporting Information Table S4.

### 7.3. Stress Effect Evaluation

Post hoc comparisons following ANOVA revealed that on the SSAI scale, the baseline (t0) was significantly lower than the stress (t1) level (*t*[36] = −10.641, *p* < 0.05]) and the post-stress recovery (t2) level was significantly lower than the stress (t1) level (*t*[36] = 12.661, *p* < 0.05). There was no significant difference between the baseline (t0) and the post-stress recovery (t2) level (*t*[36] = 0.179, *p* > 0.05). On the PANAS-N scale, the baseline (t0) level was significantly higher than the post-stress recovery (t2) level (*t*[36] = 3.849, *p* < 0.05), and the other comparisons yielded comparable findings (Table 2). These findings from both scales suggest that the stress induction and recovery processes were effective.

**Figure 2:**
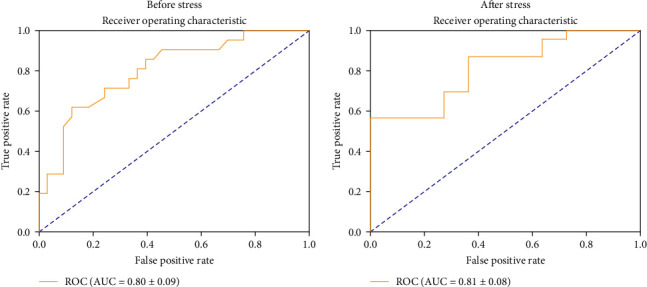
Comparison of ROC curve before and after stress.

Phase 2 results (Table 3) showed that the model trained on data collected after the stress induction (A01) demonstrated superior performance, achieving an AUC of 72.81%, an accuracy of 72.38%, and an F1 score of 0.705. Moreover, models trained on post-stress data outperformed those trained on pre-stress data.The ROC curve of A01 is shown in Figure 2.

## 8. Discussion

The present study and the findings from Phase 1 indicate that both acoustic features and word frequency features demonstrate significant potential in identification of suicide risk. Nonetheless, each feature set has distinct limitations. Acoustic features, encompassing a range of sound characteristics, are categorized into three main types: quality, prosodic, and spectral features. These features are susceptible to inaccuracies due to environmental noise and the quality of recording equipment. Moreover, individual acoustic features can vary based on physiological factors such as gender and age, which can complicate the analysis and diminish the model's ability to generalize to new data. Acoustic features may not only reflect emotional states but also other physiological factors like fatigue or illness, which could result in misclassification of suicide risk. Conversely, acoustic features are characterized by their high dimensionality, requiring substantial computational resources and posing a heightened risk of model overfitting. Previous research has focused on the detection of suicidal ideation and behavior solely through the acoustic features of speech. It has been established that there is a correlation between acoustic features and suicidal tendencies [46, 47]. For instance, individuals with suicidal tendencies tend to exhibit reduced energy and diminished pitch variation in their prosodic features, and their speech signal decay rate is typically slower. These individuals often exhibit poorer laryngeal control, leading to harsh, breathy, and sharp sounds, which are reflected in the quality features [48]. Spectral features, such as MFCCs and power spectral density (PSD), have shown potential in both identifying depression and effectively recognizing suicidal tendencies [49, 50].

Word frequency features, which denote the frequency of occurrence of specific words within a text segment or document, are a prevalent method in text analysis. Such an analysis fails to account for the contextual relationship between words, which can result in an incomplete or inaccurate interpretation of the text. The polysemy and grammatical complexity of language imply that the same word can convey different meanings across different contexts, thereby, presenting a challenge in capturing these nuances. Word frequency features are influenced by cultural and linguistic habits, which differ significantly across languages and cultural contexts, thereby, limiting the model's applicability. In limited datasets, crucial words may appear infrequently, resulting in data sparsity issues that can impact the effectiveness of model training.

Studies have indicated that analyzing the frequency and language use in electronic health records (EHRs) of patients can help compare word usage before and after suicide attempts [51]. Machine learning algorithms have been used to compare the n-gram frequencies involving third-person pronouns proximate to and distant from suicide-related content. These studies identified that the frequency of specific words and word sequences can serve as valuable indicators for identifying periods of suicide risk. When examining the linguistic features of suicidal thoughts and behaviors (STBs), researchers observed that individuals with suicidal ideation often employ more intensifiers and superlatives [26]. Suicidal behavior has been linked to an increased use of pronouns, changes in verb usage, a higher prevalence of prefixes, and multifunctional words, as well as an increase in the use of nouns and prepositions, and a decrease in the use of modifiers and numbers. These findings suggest that the frequency of specific word classes may correlate with suicide risk. Additionally, studies examining the behavioral and linguistic features between social media users who died by suicide and those without suicidal ideation, such as on platforms like Sina Weibo, have revealed that the former group displayed unique linguistic patterns, especially with a higher frequency of words related to negative emotions, death, and religion [52]. This highlights the potential significance of recognizing and understanding linguistic features in evaluating suicide risk.

In light of the strengths and weaknesses of each feature set, a combined approach that leverages both acoustic and word frequency features offers a comprehensive solution. Acoustic features delve into the emotional and tonal dimensions of speech, while word frequency features concentrate on the content and semantics of language. By integrating these two types of features, a more holistic description of the data can be provided, which enhances the model's accuracy. This combination mitigates the risk of misclassification associated with relying on a single feature type, enhancing model robustness and stability. Moreover, this dual-feature approach addressed individual variations and linguistic dependencies, thereby, improving the model's generalizability across diverse populations and settings. Furthermore, the integration of acoustic and word frequency features enables a more nuanced capture of emotional and contextual changes in speech and text, increasing the sensitivity and specificity of suicide risk identification. Despite the limitations of each individual analysis, their combined use compensates for these shortcomings, leading to a more effective suicide risk detection model.

Research suggests that depression is associated with a heightened focus on negative information [53, 54]. According to the hypothesis [55], individuals with depression interpret neutral and ambiguous stimuli through themes of loss, failure, worthlessness, and rejection. Stress can activate dysfunctional schemas, leading to negative automatic thoughts centered on the cognitive triad: pessimistic views of the self, world, and future. This sensitivity to negative stimuli is particularly pronounced in those with elevated depression and suicide risk [53]. Negative materials thus effectively simulate high-stress situations, facilitating accurate assessments of emotional and cognitive states. Such emotional reactivity is linked to the development and maintenance of psychopathological conditions [56]. Using negative materials enhances the realism of simulated stress scenarios, improving data quality and the model's accuracy in identifying suicide risk.

In Phase 2, stress factors were introduced to access their impact on suicide risk identification. The differential activation theory of suicide posits that cognitive functions in individuals at risk for suicide are not different from those in the general population under normal conditions [57]. However, in the presence of significant stress events or emotional fluctuations, cognitive patterns related to suicide become activated. In essence, during stress, the emotions evoked can repeatedly trigger cognitive processes associated with suicide, thereby, intensifying suicidal ideation. The stress-vulnerability model also posits that stress factors are significantly correlated with suicidal ideation, highlighting the influence of stressful life events [58]. This model suggests that individuals susceptible to suicide tend to display irritability, fearfulness, and anger under stress, making them more susceptible to suicidal behavior than the general population.

Drawing from these theories, stress factors are pivotal in the identification of suicide risk. In the present study, post-stress speech data more accurately depict participants' psychological states under high pressure, making linguistic and acoustic features more pronounced and distinctive. This yields additional insights into suicide risk. Specifically, under stress, speech may exhibit heightened emotional fluctuations and signs of pressure. For instance, voice intensity often rises, pitch may elevate or become more variable, word duration may either extend or diminish, and the position and bandwidth of formant frequencies may shift [59]. Corresponding evidence indicates that during the Montreal imaging stress task (MIST), the fundamental frequency (F0) of speech noticeably increases, signifying an increased rate of vocal fold vibration [60]. Concurrently, speech rate may quicken, and speech segment length may expand, indicating individuals' efforts to articulate themselves more rapidly under pressure. This evidence suggests that under stress, individuals may speak faster due to nervousness and anxiety, or slower due to depression and a sense of helplessness. Changes in speech rate not only mirror the current emotional state but may also indicate underlying anxiety and despair.

Under stress, individuals exhibit altered word processing behaviors [61]. Post-stress individuals tend to process emotionally valenced information more intensely, with research showing that under negative stress conditions, attention is more rapidly diverted from negative words compared to positive or neutral words. The TSST enhances recall performance for emotional words while diminishing recall for neutral words, suggesting that the emotional valence of stimulus material influences word processing [62, 63]. This observation indirectly reflects individuals' cognitive and emotional patterns under high-pressure condition. Individuals may use words such as “desperate,” “helpless,” and “hopeless” more frequently, revealing their inner negative emotions and sense of hopelessness. The frequent use of absolute words such as “always” and “completely” indicates cognitive extremism and absolutism, which are significant indicators of suicide risk.

Post-stress speech data offer a more comprehensive insight into emotional and linguistic features, shedding light on individuals' psychological reactions and behavioral patterns under heightened pressure. This data is pivotal in the development of precise and effective suicide risk identification models. By incorporating stress factors, the dual-feature model demonstrates enhanced accuracy and sensitivity in identifying suicide risk. This underscores the significance of stress factors in suicide risk identification and highlights the necessity of exploring new contexts for using speech to identify suicide risk.

Across various contexts, such as outpatient consultations, psychotherapy sessions, or crisis interventions, the prediction of individual behavior remains a formidable challenge. Therefore, the integration of negative interview materials with dual-feature models that incorporate word frequency and acoustic features, along with stress factors, enables the simulation of real-life scenarios. This integrated approach aims to develop an accurate identification model, providing a more comprehensive perspective in speech analysis. The objective is to identify potential high-risk individuals effectively in clinical settings, facilitating early detection and intervention, thereby, mitigating irreversible outcomes.

## 9. Limitations

Despite the advancements in the research, there are several limitations that warrant consideration. Primarily, the limited sample size restricts the generalizability of the findings. The current sample may not fully represent the broader population, necessitating an increase in sample size in future studies to bolster statistical significance and enhance the generalizability of the results. Moreover, although previous studies have consistently highlighted gender as a significant factor influencing suicide risk, indicating that females tend to exhibit higher rates of suicidal ideation and attempts. However, the present study did not find a statistically significant difference in suicide risk between male and female participants. This may be attributed to the relatively small sample size, which limits the power to detect such differences. To address this, future research should consider recruiting larger, more diverse samples across multiple centers. This would enable stratified analyses based on gender and support the development of gender-specific models. Specifically, separate classification models could be trained for male and female participants to explore potential differences in acoustic and linguistic features associated with suicide risk. Such an approach may reveal distinct patterns that are obscured in aggregated analyses and could ultimately enhance the precision and effectiveness of suicide risk detection tools. A further limitation arises from the feature selection process. Although the study's focus was on word frequency and acoustic features, other potentially valuable speech analysis features, such as emotional characteristics, were not extensively examined. Expanding the range of speech and psychological features in future studies may improve the predictive capabilities of the model. The stress experiment design also presents additional challenges. Although the TSST is a standard paradigm, its applicability and feasibility across diverse contexts necessitate further validation. Exploring various stress induction methods could enrich the diversity and practicality of the experimental design. Furthermore, the potential emotional fluctuations induced by negative materials need careful consideration. In practical applications, such materials could provoke strong emotional reactions in participants, potentially impacting their psychological well-being. Striking a balance between the use of negative materials and participants' emotional stability is crucial for ensuring the ethicality and safety of the experiments. Finally, the integration of longitudinal data collection and analysis could provide valuable insights into the long-term effects of stress on suicide risk. This approach contributes to a deeper understanding of the dynamic changes of stress reactions and their sustained influence on suicide risk, offering more in-depth theoretical insights.

## 10. Conclusion

To address the significant gap in the detection of suicide risk, particularly among individuals with mental health disorders, this study explored the application of speech data analysis, with a specific emphasis on the emotional valence of question-answer materials and the integration of acoustic and word frequency features under stress-inducing conditions. The findings indicate that dual-feature model, combining acoustic and word frequency features, significantly improves the accuracy of suicide risk identification, and hold significant implications for the development of early intervention strategies within clinical practice. Despite limitations in sample size and feature selection, the findings underscore the potential of speech analysis as a valuable tool in suicide prevention efforts, highlighting the necessity for further research to substantiate and broaden these findings.

## Figures and Tables

**Table 1:** Results of the three models.

Features	Emotional valences	Recall (%)	Precision (%)	AUC (%)	Accuracy (%)	F1 score
Acoustic features	Positive	71.38	75.30	81.82	74.18	0.733
	Neutral	70.90	80.39	82.32	75.82	0.754
	Negative	71.95	75.75	75.18	74.00	0.738

Word frequency features	Positive	64.25	65.31	70.37	65.47	0.648
	Neutral	68.59	69.39	76.47	69.70	0.690
	Negative	64.63	66.06	68.71	66.18	0.653

Dual-feature	Positive	73.88	77.80	80.88	76.00	0.758
	Neutral	68.98	76.64	82.68	74.00	0.726
	Negative	**75.55**	**79.75**	**80.16**	**77.82**	**0.776**

*Note*: Bold values indicate the highest accuracy achieved in that condition.

**Table 2:** Comparison of stress effects at different stages.

Scale	F score	ta	tb	tc
SSAI	94.351***⁣*^*∗∗∗*^**	−10.641***⁣*^*∗∗∗*^**	12.661***⁣*^*∗∗∗*^**	0.179
PANAS-N	46.782***⁣*^*∗∗∗*^**	−4.988***⁣*^*∗∗∗*^**	11.700***⁣*^*∗∗∗*^**	3.849***⁣*^*∗∗∗*^**

*Note*: ta: Post hoc comparison between baseline level t0 and stress effect t1. tb: Post hoc comparison between stress effect t1 and post-stress relaxation t2. tc: Post hoc comparison between baseline level t0 and post-stress relaxation t2.

*⁣* ^*∗∗∗*^ *p* < 0.001.

**Table 3:** Results of bidirectional feature model before and after stress.

Stress condition	Runs	Recall (%)	Precision (%)	AUC (%)	Accuracy (%)	F1 score
Before stress	B01	64.47	66.79	75.08	66.76	0.656
	B02	65.39	66.45	65.02	68.29	0.659

After stress	**A01**	**69.61**	**71.44**	**72.81**	**72.38**	**0.705**
	A02	67.97	70.11	69.12	70.95	0.690

*Note*: Bold values indicate the highest accuracy achieved under that condition.

## Data Availability

The data that support the findings of this study are available upon request from the corresponding author. The data are not publicly available due to privacy or ethical restrictions.

## Supporting Information

Supporting Information Materials S1 and S2. Question–answer materials used in Phase 1 and Phase 2 of data collection to elicit speech responses from participants under structured conditions. Tables S1 and S2. Group comparisons in Phase 1, including demographic characteristics (Table S1, *n*/%) and psychological scale scores (Table S2, mean ± standard deviation). Tables S3 and S4. Group comparisons in Phase 2, including demographic characteristics (Table S3, *n*/%) and psychological scale scores (Table S4, mean ± standard deviation).
