# Supplementary material for: A Machine Learning-Based Case–Control Study on Suicide Risk Identification: Integrating Acoustic and Linguistic Features Under Stress Conditions
Source: Depress Anxiety. 2025 Aug 8;2025:1671972. doi: 10.1155/da/1671972 (PMC12356671; doi:10.1155/da/1671972)
Supplement: Supporting Information — Materials S1 and S2. Question–answer materials used in Phase 1 and Phase 2 of data collection to elicit speech responses from participants under structured conditions. Tables S1 and S2. Group comparisons in Phase 1, including demographic characteristics (Table S1, n/%) and psychological scale scores (Table S2, mean ± standard deviation). Tables S3 and S4. Group comparisons in Phase 2, including demographic characteristics (Table S3, n/%) and psychological scale scores (Table S4, mean ± standard deviation). [file 1671972.f1.docx]

**Supplementary materials**

Supplementary materials description:

sMaterials 1-2. Question-Answer materials used in Phase 1 and Phase 2 of data collection to elicit speech responses from participants under structured conditions.

sTables 1-2. Group comparisons in Phase 1, including demographic characteristics (sTable 1, n/%) and psychological scale scores (sTable 2, mean ± standard deviation).

sTables 3-4. Group comparisons in Phase 2, including demographic characteristics (sTable 3, n/%) and psychological scale scores (sTable 4, mean ± standard deviation).

sMaterials 1. Phase 1 Question-Answer Materials

sMaterials 2. Phase 2 Question-Answer Materials

sTable 1. Phase 1 comparison of demographic between groups [n(%)]

sTable 2. Phase 1 comparison of scale data between groups(x̄±s)

sTable 3. Phase 2 comparison of demographic between groups [n(%)]

sTable 4. Phase 2 comparison of scale data between groups(x̄±s)

sMaterials 1. Question-Answer Materials

| Question | pleasure | | arousal | |
| --- | --- | --- | --- | --- |
|  | average | standard | average | standard |
| Q1: If you were to suffer a major accident (such as an earthquake or car accident), and all your family members passed away, leaving only you alive, how would you feel? What impact would it have on your life? | 1.55 | 1.28 | 7.45 | 2.41 |
| Q2: If you were misunderstood and wrongly accused by a good friend, how would you feel? What would you do? | 2.81 | 1.90 | 6.06 | 2.31 |
| Q3: Under what circumstances would you feel hopeless? What would you do when you feel hopeless? | 2.68 | 2.15 | 6.00 | 2.40 |
| Q4: How do you plan your life for the next three years? | 5.74 | 1.67 | 6.02 | 1.58 |
| Q5: Please introduce your hometown. | 6.72 | 1.46 | 6.34 | 1.89 |
| Q6: What kind of person do you think you are? Please evaluate yourself from all aspects. | 6.19 | 1.48 | 5.98 | 1.48 |
| Q7: Share with us your most beautiful memories, and briefly describe the scene at that time. | 7.70 | 1.28 | 7.11 | 1.81 |
| Q8: What do you usually do when you are with your good friends? How do you feel? | 7.74 | 0.97 | 7.04 | 1.43 |
| Q9: Please briefly describe an experience that makes you feel proud. | 7.47 | 1.21 | 6.81 | 1.64 |

sMaterials 2. Phase 2 Question-Answer Materials

| Question | pleasure | | arousal | |
| --- | --- | --- | --- | --- |
|  | average | standard | average | standard |
| Q1: If you were to suffer a major accident (such as an earthquake or car accident), and all your family members passed away, leaving only you alive, how would you feel? What impact would it have on your life? | 1.55 | 1.28 | 7.45 | 2.41 |
| Q2: If you were misunderstood and wrongly accused by a good friend, how would you feel? What would you do? | 2.81 | 1.90 | 6.06 | 2.31 |
| Q3: Under what circumstances would you feel hopeless? What would you do when you feel hopeless? | 2.68 | 2.15 | 6.00 | 2.40 |
| Q4: If the person you love the most has passed away, how would you react? Imagine what impact it would have on your life. | 1.68 | 1.53 | 7.17 | 2.59 |

sTable 1. Phase 1 comparison of demographic between groups [n(%)]

| Variable |  | Low Suicide Risk Group（n=38） | High Suicide Risk Group（n=22） | *t/χ^2^* Value | *P* Value |
| --- | --- | --- | --- | --- | --- |
| Age | mean ± SD | 22.45±2.60 | 21.77±1.85 | 1.169 | 0.247 |
| Gender | Male | 12（31.6） | 8（36.4） | 0.144 | 0.705 |
|  | Female | 26（68.4） | 14（63.6） |  |  |
| Only Child | Yes | 19（50.0） | 11（50.0） | 0.000 | 1.000 |
|  | No | 19（50.0） | 11（50.0） |  |  |
| Marital Status | Single | 24（63.2） | 13（59.1） | 0.97 | 0.755 |
|  | Non-single | 14（36.8） | 9（40.9） |  |  |
| Family Residence | Rural | 7（18.4） | 1（4.5） | 3.853 | 0.278 |
|  | Small Town or County | 8（21.1） | 6（27.3） |  |  |
|  | Medium or Large City | 10（26.5） | 4（18.2） |  |  |
|  | Large City | 13（34.2） | 11（50.0） |  |  |
| Family Economic Status | Good | 7（18.4） | 8（36.4） | 2.358 | 0.308 |
|  | Average | 25（65.8） | 11（50.0） |  |  |
|  | Bad | 6（15.8） | 3（13.6） |  |  |
| Family History of Mental Illness | Yes | 33（86.8） | 17（77.3） | 0.359 | 0.549 |
|  | No | 5（13.2） | 5（22.7） |  |  |
| Family History of Suicide | Yes | 34（89.5） | 18（81.8） | 0.199 | 0.655 |
|  | No | 4（10.5） | 4（18.2） |  |  |
| Acquaintance Suicide History | Yes | 33（86.8） | 16（72.7） | 1.031 | 0.310 |
|  | No | 5（13.2） | 6（27.3） |  |  |

sTable 2. Phase 1 comparison of scale data between groups(‾x±s)

| Variable | Low Suicide Risk Group | High Suicide Risk Group | *t* Value | *P* Value |
| --- | --- | --- | --- | --- |
| PHQ-9 | 10.24±6.01 | 15.64±4.71 | 3.616 | <0.001 |
| SBQ-R | 9.87±2.55 | 13.73±2.66 | 5.564 | <0.001 |
| BSI-CV | 13.84±7.00 | 22.05±7.13 | 4.344 | <0.001 |
| YMRS | 0.36±0.79 | 0.31±1.07 | 0.183 | 0.856 |

*p<0.05，**p<0.01，***p<0.001

sTable 3. Phase 2 comparison of demographic between groups [n(%)]

| Variable |  | Low Suicide Risk Group（n=12） | High Suicide Risk Group（n=26） | t/χ^2^ Value | P Value |
| --- | --- | --- | --- | --- | --- |
| Age | mean ± SD | 20.92±3.73 | 20.07±5.51 | 0.558 | 0.581 |
| Gender | Male | 3（25） | 8（30.8） | 0.133 | 0.715 |
|  | Female | 9（75） | 18（69.2） |  |  |
| Only Child | Yes | 2（16.7） | 8（30.8） | 0.272 | 0.602 |
|  | No | 10（83.3） | 28（69.2） |  |  |
| Marital Status | Single | 7（58.3） | 17（65.4） | 0.779 | 0.377 |
|  | Non-single | 5（41.7） | 9（34.6） |  |  |
| Family Residence | Rural | 3（25.0） | 3（11.5） | 1.614 | 0.204 |
|  | Small Town or County | 4（33.3） | 5（19.2） |  |  |
|  | Medium or Large City | 1（8.3） | 7（26.9） |  |  |
|  | Large City | 4（33.3） | 11（42.3） |  |  |
| Family Economic Status | Good | 6（50.0） | 7（26.9） | 1.048 | 0.306 |
|  | Average | 4（33.3） | 14（53.8） |  |  |
|  | Bad | 2（16.7） | 5（19.2） |  |  |
| Family History of Mental Illness | Yes | 2（16.7） | 1（3.8） | 1.700 | 0.192 |
|  | No | 10（83.3） | 25（96.2） |  |  |
| Family History of Suicide | Yes | 0 | 0 |  |  |
|  | No | 12（100） | 26（100） |  |  |
| Acquaintance Suicide History | Yes | 2（16.7） | 7（26.9） | 0.079 | 0.779 |
|  | No | 10（83.3） | 19（73.1） |  |  |

sTable 4. Phase 2 comparison of scale data between groups(‾x±s)

| Variable | Low Suicide Risk Group | High Suicide Risk Group | *t* Value | *P* Value |
| --- | --- | --- | --- | --- |
| PHQ-9 | 12.58±5.544 | 18.73±4.75 | 3.547 | 0.0011**^**^** |
| SBQ-R | 6.27±2.35 | 12.81±3.09 | -11.52 | >0.001 |
| BSI-CV | 12.422±6.16 | 20.77±7.04 | 3.527 | 0.0012**^**^** |
| YMRS | 1.25±2.83 | 1.73±2.71 | 0.502 | 0.619 |

*p<0.05，**p<0.01，***p<0.001
